# CircMETTL3, upregulated in a m6A-dependent manner, promotes breast cancer progression

**DOI:** 10.7150/ijbs.57783

**Published:** 2021-03-15

**Authors:** Zhi Li, Hai-Yan Yang, Xin-Yuan Dai, Xu Zhang, Yu-Zhou Huang, Liang Shi, Ji-Fu Wei, Qiang Ding

**Affiliations:** 1 Jiangsu Breast Disease Center, the First Affiliated Hospital with Nanjing Medical University, 300 Guangzhou Road, Nanjing 210029, Jiangsu Province, P.R. China.; 2 Research Division of Clinical Pharmacology, the First Affiliated Hospital with Nanjing Medical University, 300 Guangzhou Road, Nanjing 210029, Jiangsu Province, P.R. China.

**Keywords:** circMETTL3, miR-31-5p, CDK1, N6-methyladenosine, breast cancer

## Abstract

Growing evidence indicates N6-methyladenosine (m6A) has biological function in oncogenesis. METTL3, the catalytic component, is the most important part of methyltransferase complex and plays a crucial role in cancers. However, the biological function of circRNAs derived from *METTL3* in breast cancer and the underlying molecular mechanism remains unclear. Herein, we report circMETTL3, which has not been explored in breast cancer, and it is markedly upregulated in breast cancer. Moreover, we uncovered that circMETTL3 could facilitate cell proliferation, migration and invasion in breast cancer. Mechanism investigation showed that circMETTL3 might act as a competing endogenous RNA (ceRNA) of miR-31-5p and upregulate its target cyclin-dependent kinases (CDK1). Moreover, m6A modification of circMETTL3 might affect its expression. Taken together, our results elucidate that circMETTL3 promotes breast cancer progression through circMETTL3/miR-31-5p/CDK1 axis. Moreover, METTL3, the host gene of circMETTL3, may regulate circMETTL3 expression in an m6A-dependent manner, while circMETTL3 has no effect on METTL3 expression, providing a new relationship between the circRNA and the corresponding host gene. Thus, it may serve as a new therapeutic target for breast cancer.

## Introduction

Breast cancer is the most commonly diagnosed malignancy and most frequent cause of cancer-related mortality in women 1, 2. Despite the outstanding advances in the diagnosis and treatment of breast cancer in recent decades, the morbidity and mortality are still high, due to high frequency of metastasis, recurrence, and drug resistance. Therefore, it is necessary to further study the molecular mechanism and develop more efficient and precise targeting therapies to treat breast cancer.

Circular RNAs (circRNAs) are a subgroup of non-coding RNAs and covalently closed loops formed by back-splicing. They are highly conserved and stable due to the lack of 5'-cup structure and 3'-polyadenylated tail 3-5. It has been demonstrated that circRNAs are produced by a non-canonical alternative splicing, and RNA-binding proteins or transcription factor facilitate the generation of circRNAs 6, 7. Growing studies showed that circRNAs played a critical role in various biological processes in cancers, including proliferation, metastasis and autophagy, via acting as a sponge of miRNAs, interacting with RNA binding proteins or encoding peptides 8-10. In breast cancer, many circRNAs were found to play an important role in tumor progression 11-13. For example, circFBXW7 inhibited breast cancer by sponging miR-197-3p and encoding a 185-aa protein 13. circSMARCA5 inhibited DNA damage repair by interacting with host gene 14.

N6-methyladenosine (m6A) methylation, one of the most prevalent modifications in RNA, affects RNA splicing, stability, translation 15, 16. m6A modification is a dynamic process, controlled by methyltransferase enzymes (including METTL3, METTL14, WTAP, KIAA1429 *et al*.) and demethylase enzymes (ALKBH5 and FTO) 17. Previous studies showed these proteins played important roles in breast cancer developments 18-22, especially METTL3, which is the key component in methyltransferase enzymes 20, 23-25. For example, METTL3 could promote breast cancer progression via inhibiting tumor suppressor let-7g or targeting Bcl-2 20, 25, though another study showed METTL3 inhibited metastasis of triple-negative breast cancer by decreasing COL3A1 24. METTL3 also increased adenylate kinase 4 expression, causing the resistance of breast cancer cells to tamoxifen 23. A recent interesting study displayed circ_KIAA1429 derived from *KIAA1429*, a part of methyltransferase complex, could accelerate hepatocellular carcinoma advancement through the mechanism of m6A-YTHDF3-Zeb1 26. We then analyzed the expression profiles of circRNAs in breast cancer tissues by analyzing GEO database (GSE113230) and characterized a novel circRNA derived from the METTL3 gene, termed as circMETTL3. This circRNA is a 623 nt transcript originated from the exon-2 and exon-3 of METTL3 gene. However, the function of circMETTL3 in breast cancer is still unknown.

In this study, we found circMETTL3 was upregulated in breast cancer tissues and cell lines in an m6A-dependent manner. It could promote breast cancer proliferation and metastasis. Mechanistically, circMETTL3 sponged miR-31-5p to upregulate CKD1 expression. Our findings suggested that circMETTL3 might serve as a potential biomarker and therapeutic target for breast cancer patients.

## Materials and Methods

### Clinical samples

Breast cancer tissues and adjacent normal tissues were obtained from the First Affiliated Hospital with Nanjing Medical University. All patients received no neoadjuvant therapy. The collected samples were frozen in liquid nitrogen immediately after resection. All patients provided written informed consent, and the study was approved by the Ethics Committee of the First Affiliated Hospital with Nanjing Medical University.

### Cell culture

Human breast cancer cell lines (MCF-7, ZR-75-1, BT474, MDA-MB-231, MDA-MB-453 and HCC1806) and normal mammary epithelial cell line (MCF-10A) were purchased from American Type Culture Collection (ATCC) (Manassas, VA, USA). SUM1315 was kindly provided by Stephen Ethier (University of Michigan, AnnArbor, MI, USA). 293T cell line was preserved by our lab. MCF-7, ZR-75-1, BT474, MDA-MB-231, MDA-MB-453, SUM1315 and 293T cells were cultured in DMEM (Gibco, USA), HCC1806 cells were cultured with RPMI 1640 (Gibco, USA), containing 10% fetal bovine serum, 100 U/mL penicillin and 100 mg/mL streptomycin. Cells in the medium were incubated in a humidified atmosphere containing 5% CO_2_ at 37 °C.

### RNA extraction and quantitative real time polymerase chain reaction (qRT-PCR)

Total RNA from cells or tissues was isolated using Trizol reagent (Takara, Japan). Complementary DNA (cDNA) was synthesized using the HiScript Q RT SuperMix (Vazyme, China). Then, qRT-PCR was performed using AceQ qPCR SYBR Green Master Mix (Vazyme, China). For miRNA expression analysis, cDNA was specifically synthesized and miRNA was detected with Bulge-Loop™ miRNA qRT-PCR (Ribobio, China). β-actin and U6 were used as the endogenous control for the relative expression of mRNA and miRNA respectively. The specific primers used are listed in Table S1. The relative expression was calculated by the 2^-ΔΔCT^ method.

### Actinomycin D and RNase R treatment

Transcription was prevented by the addition of 2 mg/mL actinomycin D or DMSO (Sigma-Aldrich, USA) as the negative control to the cell culture medium. After treatment in the indicated time, the RNA expression levels of circMETTL3 and METTL3 were detected by qRT-PCR. For RNase R treatment, the original RNAs extracted from ZR-75-1 or SUM1315 cells were divided into two equal parts, one for RNase R treatment (RNase R) and the other for non-treatment (mock). A total of 2 mg RNA was incubated for 30 min at 37 °C with or without 3 U/mg RNase R (Epicenter Technologies, USA). The internal reference (β-actin) in the mock group was used as the calculation standard.

### Cell transfection

Transfections were carried out using the Lipofectamine 3000 Reagent (Invitrogen, USA) following the manufacturer's protocol. The small interfering RNAs (siRNAs) targeting circRNA were designed and synthesized by Ribobio (Guangzhou, China). miRNA mimics, miRNA inhibitors and their negative controls were designed and synthesized by GenePharma (Shanghai, China). Commercially available lentiviral vectors containing circMETTL3 sequence was constructed by Hanheng (Shanghai, China). Stable cells were selected using puromycin 5 μg/mL. The sequences of siRNAs and RNA oligonucleotides were listed in Table S2.

### Cell proliferation assay

Cell proliferation assays were conducted by using Cell Counting Kit-8 (CCK-8) (Vazyme, China) in accordance with the manufacturer's instructions. 2000 cells were seeded into each well of 96-well plate with 100 μL DMEM supplemented with 10% FBS. At the indicated time point of everyday, the medium was exchanged by 100 μL DMEM with CCK-8 (90 μL DMEM and 10 μL CCK-8) and the cells were incubated for 2h. Absorbance was detected spectrophotometrically at 450 nm.

### Colony formation assay

A total of 500 cells were plated in a six-well plate and maintained in DMEM medium containing 10% FBS for approximately 2 weeks. Proliferating colonies were fixed with methanol and stained with 1% crystal violet (Beyotime, China). The number of colonies was counted by observing the proliferation of single cell.

### 5-ethynyl-2'-deoxyuridine (EdU) incorporation assay

Cell proliferation was measured via the EdU assay (Beyotime, China). The cells were cultured in 96-well plates (2 × 10^4^ cells/well) with DMEM (10% FBS) for 24 h, incubated with 50 μM EdU at 37 °C for 2 h, treated with 4% paraformaldehyde and 0.5% Triton X-100 and stained with 1×Apollo® reaction cocktail for 30 min. Lastly, nuclei were stained with DAPI, and the cells were visualized under a fluorescence microscope.

### Cell wound-healing assay

Cell wound-healing assay was used to evaluate cell migration. Cells were seeded in 6-well plates and cultured to the subfusion state. Then linear scratch wounds were created by 200 μL sterile pipette tip. After washing three times with PBS, images were captured by inversion fluorescence microscopy at 0 and 48 h (Olympus, Japan).

### Transwell assay

Transwell chambers with a membrane pore size of 8 mm were coated without or with Matrigel (BD Biosciences, USA). 200 μL cell suspension in serum-free DMEM was added to the upper chambers, whereas 600 μL medium containing 10% FBS was used in the lower chamber. After incubation for 24 h or 48 h, the cells were stained with crystal violet (Beyotime, China) to assess migration or invasion respectively.

### Fluorescence *in situ* hybridization (FISH)

FISH assay was executed to observe the location of circMETTL3 in breast cancer cells. Briefly, probes for circMETTL3 were synthesized with 3′-Cy3-modification (RiboBio, China), and the cells were immobilized with 4% paraformaldehyde, followed by 0.5% Triton X-100. Then cells were incubated with 200 μL of hybridization which contain corresponding probe overnight at 37 °C. After that, the cellular DNA was stained with DAPI and photographed by confocal microscope.

### Dual-luciferase reporter assay

The putative miR-31-5p binding sequences in circMETTL3 or CDK1-3ʹ-untranslated Regions(UTR) and their mutant of the binding sites were synthesized and cloned to downstream of the luciferase gene in the pmirGLO luciferase vector (Promega, USA). We then co-transfected the pmirGLO reporter gene with miR-31-5p mimics or miR-31-5p NC. The relative luciferase activity was measured with the Dual-Luciferase reporter assay system (Promega, USA).

### RNA immunoprecipitation (RIP)

An RNA immunoprecipitation (RIP) assay was conducted in breast cancer cells using the Magna RIP RNA-Binding Protein Immunoprecipitation Kit (Millipore, Billerica, MA, USA) according to manufacturer's instructions. Anti-Argonaute2 (AGO2) antibody (Abcam, USA, ab186733) or NC rabbit IgG (Cell Signaling Technology, USA, 7076P2) were used. Briefly, cells were collected and lysed by the RIP lysis buffer. Then the corresponding antibodies were added into the cleared lysates and incubated in the magnetic beads' suspension with rotating overnight at 4 °C. Precipitate was digested with proteinase K buffer, and then qRT-PCR was used to measure the levels of circMETTL3 transcripts in the AGO2 or IgG immunocomplexes.

The m6A RIP was performed as previously described with some modifications. Briefly, 3 μg of anti-m6A antibody (Abcam, USA, ab208577) was conjugated to protein A/G magnetic beads overnight at 4 °C. And then the antibody conjugated beads were incubated with the antibody in IP buffer with RNase inhibitor and protease inhibitor. The interacting RNAs were isolated and detected by qRT-PCR.

### RNA pull down assay

A total of 1 × 10^7^ breast cancer cells were harvested, lysed, and sonicated. The cell lysates were incubated with streptavidin-coated magnetic beads to pull down the biotin-labeled RNA complex at 25 °C for 2 h to generate probe-coated beads. Cell lysate with circMETTL3 probe or oligo probe was incubated at 4 °C for one night. After washing with wash buffer, the RNA mix bound to the beads was eluted and the complex was purified with Trizol (Takara, China).

### Western blot analysis

The total protein of breast cancer cells was exacted with RIPA buffer and separated by 10% SDS-PAGE, then electransferred onto a PVDF membrane (Bio-Rad, USA). The membranes were blocked with 5% skimmed milk powder and incubated with primary antibodies against METTL3 (1:1000, Abcam, USA, ab195352), CDK1 (1:1000, Proteintech, USA, 19532-1-AP), β-actin (Proteintech, China, 20536-1-AP) at 4 °C overnight and then incubated with secondary antibodies (1:5000) (Cell Signaling Technology, USA, 7074P2) at room temperature for 2h. Finally, the bands were examined by Immobilob™ Western Chemiluminescent HRP Substrate (Millipore, USA).

### Animal experiment

All animal experiments were conducted according to the guidelines of Institutional Animal Care and Use Committee of the Nanjing Medical University. Twelve female BALB/c nude mice (aged 4 weeks, 18-22g) were randomly divided into 2 groups. Stable circMETTL3-expression SUM1315 cells or control cells (1×10^6^ cells in 0.1 mL PBS) was subcutaneously injected into mammary fat pads of the mice and the growth of tumors was followed up every week. Tumor volume was measured every week using a caliper, calculated as (length × width^2^)/2. After 4 weeks, mice were sacrificed and checked for final tumor weight.

### Statistical analysis

The statistical analysis was performed by SPSS software (Version 25.0) and GraphPad Prism (Version 8.0), and presented as mean ± standard deviation (SD). All data were analyzed by two-tailed Student's t-test. Each experiment was repeated at least three times, and p < 0.05 was considered statistically significant.

## Results

### Characterization of circMETTL3 in breast cancer

**Figure 1:**
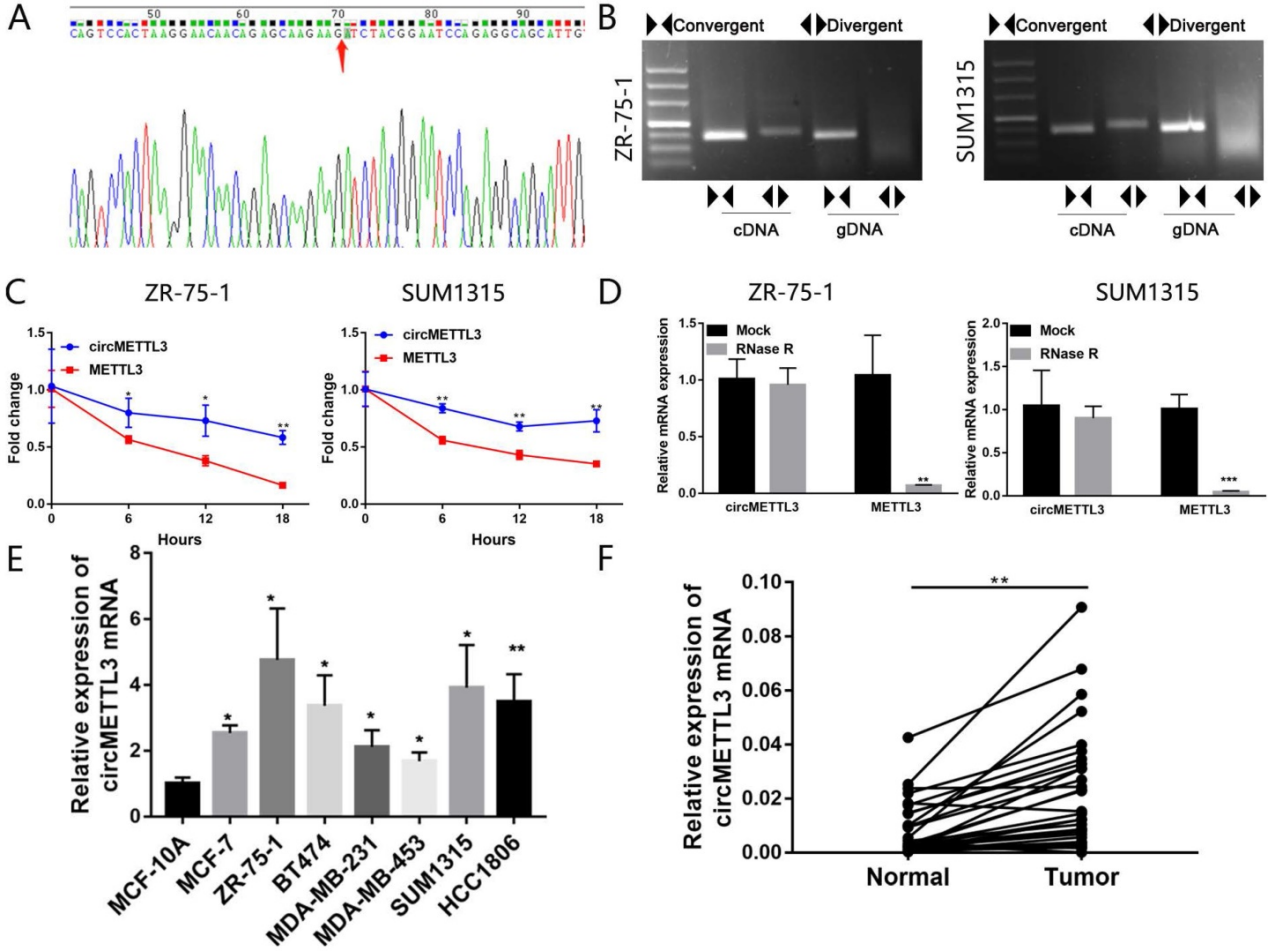
** Characterization of circMETTL3 in breast cancer cells and tissues. A.** Sanger sequencing-validated circular structure. Arrows indicated the junction site of circMETTL3. **B.** RT-PCR analysis for circMETTL3 and its linear transcript METTL3 in cDNA and gDNA in breast cancer cells. **C.** qRT-PCR analysis of circMETTL3 and METTL3 after treatment with actinomycin D at the indicated time points. **D.** qRT-PCR showed the expression of circMETTL3 and METTL3 mRNA in breast cancer cells treated with RNase R or mock control. **E.** Expression of circMETTL3 in the breast cancer cell lines and MCF-10A by qRT-PCR. F. Expression of circMETTL3 in breast cancer tissues and adjacent normal tissues. Data were shown as mean ± SD. *p <0.05, **p <0.01, ***p<0.001.

Firstly, we performed sanger sequencing to detect the junction site of circMETTL3 and found it formed the spliced mature sequence with a full length of 623 nt (Fig. 1A). We conducted PCR using cDNA and gDNA (genomic DNA) as templates with two sets of primers for circMETTL3. The convergent primers could amplify circMETTL3 using cDNA and gDNA as templates, while the divergent primers only amplified circMETTL3 using cDNA as templates, and no amplifications were observed using gDNA templates (Fig. 1B). We next investigated the stability of circMETTL3. After treatment with actinomycin D or RNase R, total RNAs were harvested from ZR-75-1 and SUM1315 cells at the indicated time points. The qRT-PCR assay showed circMETTL3 was more stable than METTL3 (Fig. 1C-D). circMETTL3 was highly expressed in breast cancer cell lines (Fig. 1E). METTL3 was also highly expressed in the same set of cell lines (Fig. S1). Moreover, it was expressed higher in breast cancer tumor tissues than adjacent normal tissues significantly (Fig. 1F). These results indicated that circMETTL3 was consistently expressed in breast cancer and might play an important role in breast cancer progression.

### circMETTL3 promotes breast cancer cell proliferation *in vitro* and *in vivo*

**Figure 2:**
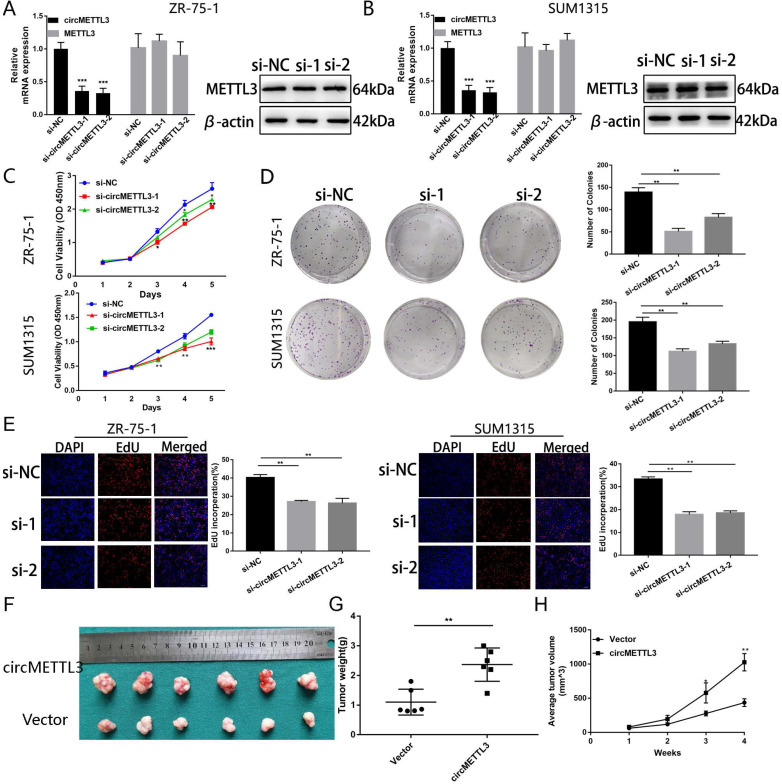
** Knockdown of circMETTL3 inhibits breast cancer cell proliferation *in vitro* and *in vivo*.** A-B. qRT-PCR and western blot analysis of the transfection efficacy of siRNA on circMETTL3 and METTL3 expression in ZR-75-1 (A) and SUM1315 (B) cell lines after 48 h transfection. C. The proliferation ability of ZR-75-1 and SUM1315 cells lines transfected with siRNAs was determined by CCK-8 assays. D. The colony formation results of ZR-75-1 and SUM1315 cells lines transfected with siRNAs. Colonies > 50 mm were counted. E. EdU assays results of ZR-75-1 and SUM1315 cells lines transfected with siRNAs. Scale bar, 100 µm. Blue indicates DAPI, red indicates EdU, si-NC indicates negative control, si-1 indicates si-circMETTL3-1,si-2 indicates si-circMETTL3-2. F. Representative images of xenograft tumors of circMETTL3 overexpression (circMETTL3) and control (Vector) group (n = 6). G. Tumor weights in these two groups were shown. H. Tumor volumes were measured weekly in all mice that received subcutaneous injections. Data were shown as mean ± SD. *p <0.05, **p <0.01, ***p<0.001.

To explore the function of circMETTL3 in breast cancer, we knocked down circMETTL3 expression with small interfering RNAs (siRNA) and found circMETTL3 expression was inhibited, without affecting the parental gene *METTL3* (Fig. 2A-B). CCK-8 assay showed that knockdown of circMETTL3 could inhibit breast cancer cell proliferation significantly (Fig. 2C), consistent with the results from colony formation assay (Fig. 2D) and EdU assay (Fig. 2E). Accordingly, overexpression of circMETTL3 could promote breast cancer cells proliferation ability (Fig. S2A-D). Moreover, overexpression of circMETTL3 could facilitate the tumorigenesis of breast cancer cells *in vivo* (Fig. 2F-H). circMETTL3 was overexpressed in the treated group compared with the control group without affecting METTL3 expression (Fig. S3A-B).

### circMETTL3 enhances breast cancer cells migration and invasion ability

**Figure 3:**
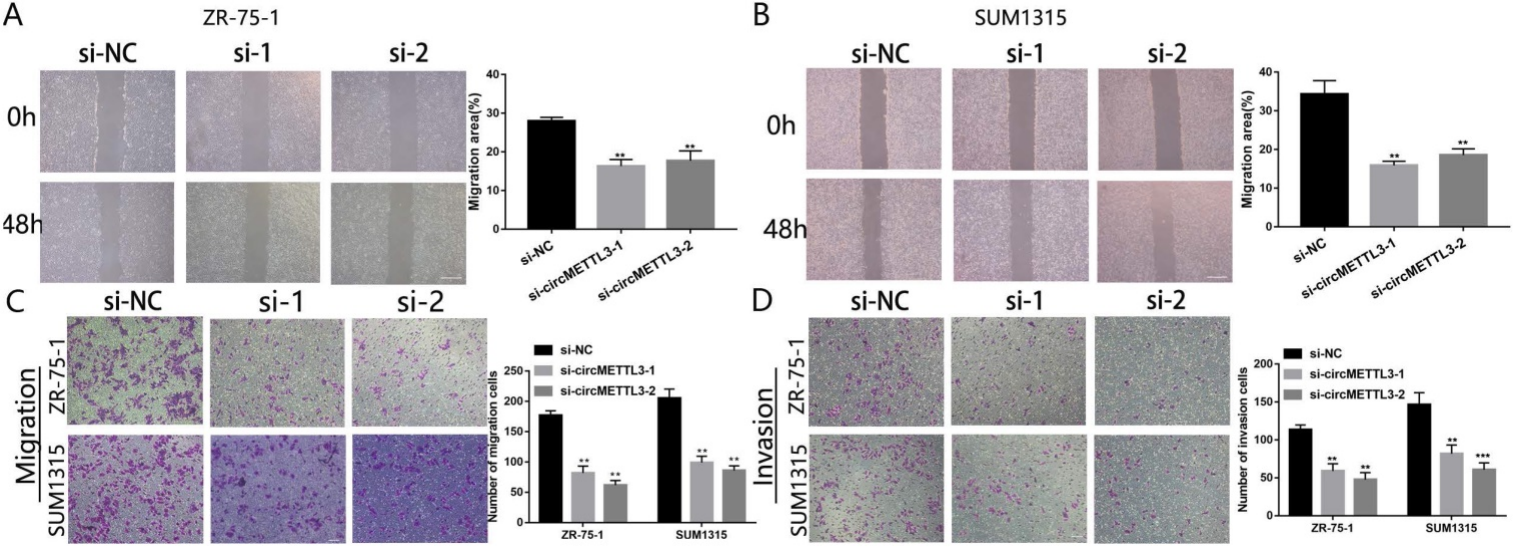
** circMETTL3 enhances breast cancer cells migration and invasion ability.** A-B. The wound healing assays were performed to assess the effect of circMETTL3 on cell motility at 0 and 48 h in ZR-75-1 (A) and SUM1315 (B) cell lines. Scale bar, 100 µm. C-D. The representative images of migrated (C) and invaded (D) ZR-75-1 and SUM1315 cell lines after circMETTL3 knockdown. Scale bar, 100 µm.si-NC indicates negative control, si-1 indicates si-circMETTL3-1,si-2 indicates si-circMETTL3-2. Data were shown as mean ± SD, *p <0.05, **p <0.01, ***p<0.001.

We next investigated the effect of circMETTL3 on breast cancer cells metastasis and invasion. The wound healing assay showed that downregulation of circMETTL3 could decrease breast cancer cells migration significantly (Fig. 3A-B); by contrast, overexpression of circMETTL3 could enhance breast cancer cells migration (Fig. S4A-C). In the Transwell assay, the number of migration cells decreased dramatically after the circMETTL3 expression was reduced (Fig. 3C). An opposite effect was detected in the breast cells when circMETTL3 was overexpressed (Fig. S4D, F). Moreover, reduced circMETTL3 expression could inhibit breast cancer cells invasion ability (Fig. 3D), but increasing circMETTL3 expression could reverse the effect (Fig. S4E, G).

### circMETTL3 acts as a sponge and targets miR-31-5p directly

**Figure 4:**
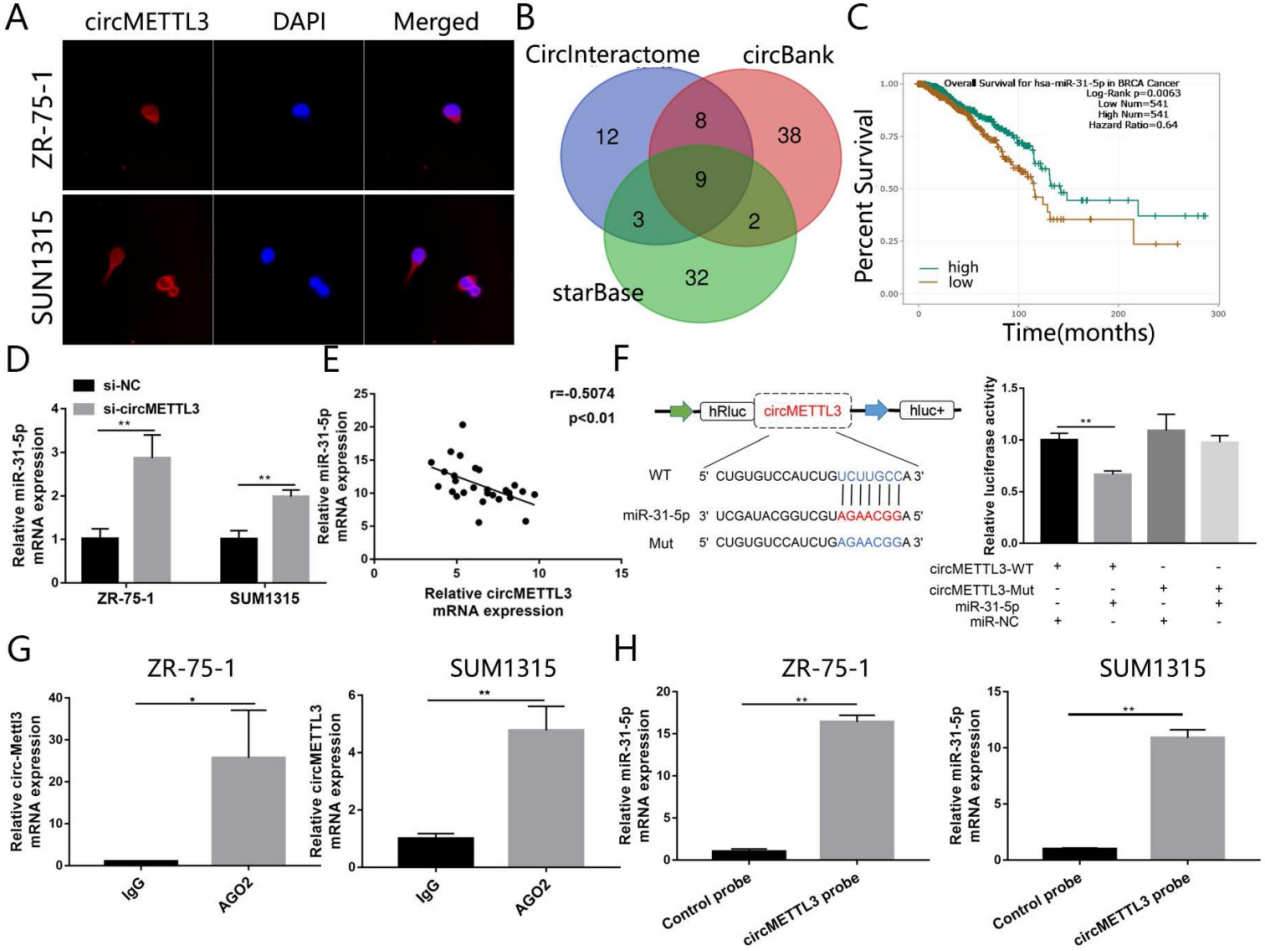
** circMETTL3 acts as the sponge of miR-31-5p in breast cancer.** A. FISH revealed the subcellular localization of circMETTL3. Red indicates circMETTL3, bule indicates DAPI. B. The Venn-diagram showed the potential miRNAs sponged by circMETTL3 predicted by combination of three bioinformatics algorithms (CircInteractome, circBank, starBase). C. Kaplan-Meier survival analysis showed the correlation between the expression of miR-31-5p and the overall survival of breast cancer patients based on TCGA. D. Knockdown of circMETTL3 increased expression of miR-31-5p in breast cancer cell lines. E. Correlation between circMETTL3 and METTL3 expression in breast cancer tissues. F. Schematic representation of circMETTL3-WT and circMETTL3-Mut luciferase reporter vectors (left). Luciferase reporter assay indicated the binding capacity of circMETTL3 with miR-31-5p (right). G. Anti-AGO2 RIP experiments were performed to detect circMETTL3 expression in breast cancer cell lines. ZR-75-1 and SUM1315 cells lysates were immunoprecipitated with AGO2 or IgG antibody and analyzed by using qRT-PCR to detect circMETTL3 transcript levels. H. RNA pull-down was performed to detect the enrichment of miR-31-5p in breast cancer cell lines. Data were shown as mean ± SD, *<0.05, **p <0.01, ***p<0.001.

To dissect the underlying mechanism by which circMETTL3 promotes breast cancer proliferation and metastasis, we performed FISH assay to observe the subcellular localization of circMETTL3. The results showed circMETTL3 was mainly located in cytoplasm in breast cancer cells (Fig. 4A), suggesting circMETTL3 might act as miRNA sponge. Through bioinformatics analysis, we took the intersection of the miRNAs predicted in CircInteractome with context+ score percentile greater than 90, the circBank and starBase predictions (Fig. 4B), and combined the correlation between and the miRNAs expression and breast cancer patients prognosis. miR-31-5p and miR-769-5p might be sponged by circMETTL3. But knockdown of circMETTL3 had no effect on miR-769-5p expression. We identified miR-31-5p as the candidate sponged by circMETTL3. Survival curve showed that patients with high expression of miR-31-5p had better overall survival rate (Fig. 4C). Moreover, knockdown of circMETTL3 could increase miR-31-5p expression in breast cancer cells (Fig. 4D) while overexpression of circMETTL3 decreased miR-31-5p expression in the previous animal tumors (Fig. S5A). Moreover, we detected correlation between miR-31-5p and circMETTL3 expression in breast cancer tissues and found the expression of miR-31-5 and circMETTL3 is negatively correlated (Fig. 4E). Then, dual-luciferase reporter assays were performed to measure the binding capacity between circMETTL3 and miR-31-5p in breast cancer cells. The wild and mutant dual-luciferase reporter plasmids of circMETTL3 were constructed accordingly. The results indicated that miR-31-5p mimics reduced the luciferase activity of circMETTL3-WT luciferase reporter obviously, but not that of mutants (Fig. 4F). It is well known that miRNAs degrade mRNAs and inhibit translation in an AGO2-dependent manner via binding to their targets directly. We thus performed RIP assay and found that circMETTL3 was enriched in AGO2 immunoprecipitates in breast cancer cells (Fig. 4G). Furthermore, we performed pull down assay and found that miR-31-5p was abundantly pulled down by circMETTL3 probe, compared with negative control probe in breast cancer cells (Fig. 4H). Overall, these results suggested that circMETTL3 could act as a sponge and targets miR-31-5p directly.

### miR-31-5p partly reverses the effects caused by circMETTL3 in breast cancer cells

**Figure 5:**
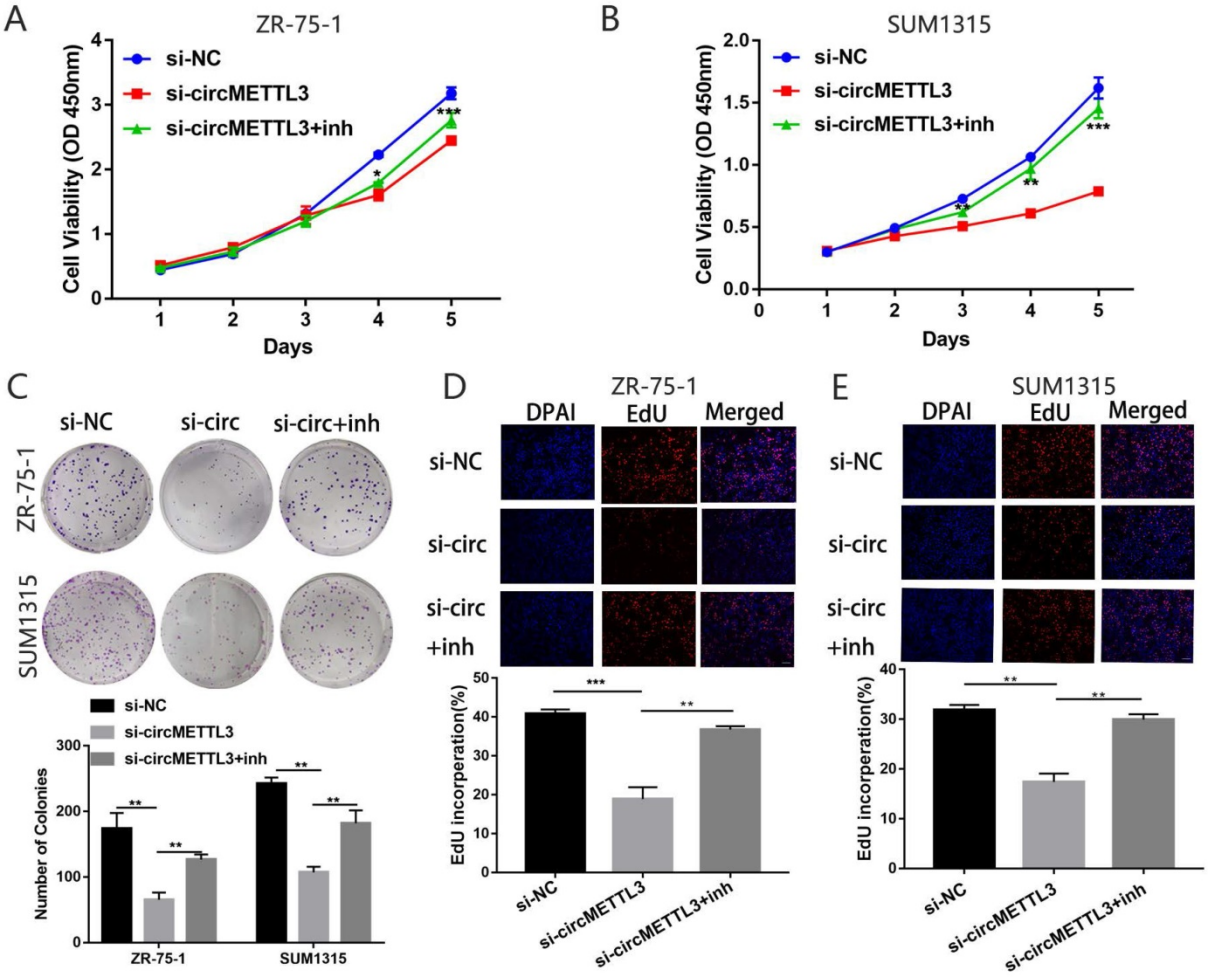
** miR-31-5p partly reverses the cell proliferation caused by circMETTL3 in breast cancer cells lines.** A-B. Cell proliferation of ZR-75-1 (A) and SUM1315 (B) cell lines transfected with negative control (si-NC), si-circMETTL3 (si-circ) or circMETTL3+miR-31-5p inhibitor (si-circMETTL3+inh) was determined by CCK-8. C: Cell proliferation of ZR-75-1 and SUM1315 cell lines transfected with negative control, si-circMETTL3 or circMETTL3+miR-31-5p inhibitor was determined by colony formation assay. Colonies > 50 mm were counted. D-E. Cell proliferation of ZR-75-1 (D) and SUM1315 (E) cell lines transfected with negative control, si-circMETTL3 or circMETTL3+miR-31-5p inhibitor. Blue indicates DAPI, red indicates EdU.Scale bar, 100 µm. Data were shown as mean ± SD, *p <0.05, **p <0.01, ***p<0.001.

**Figure 6:**
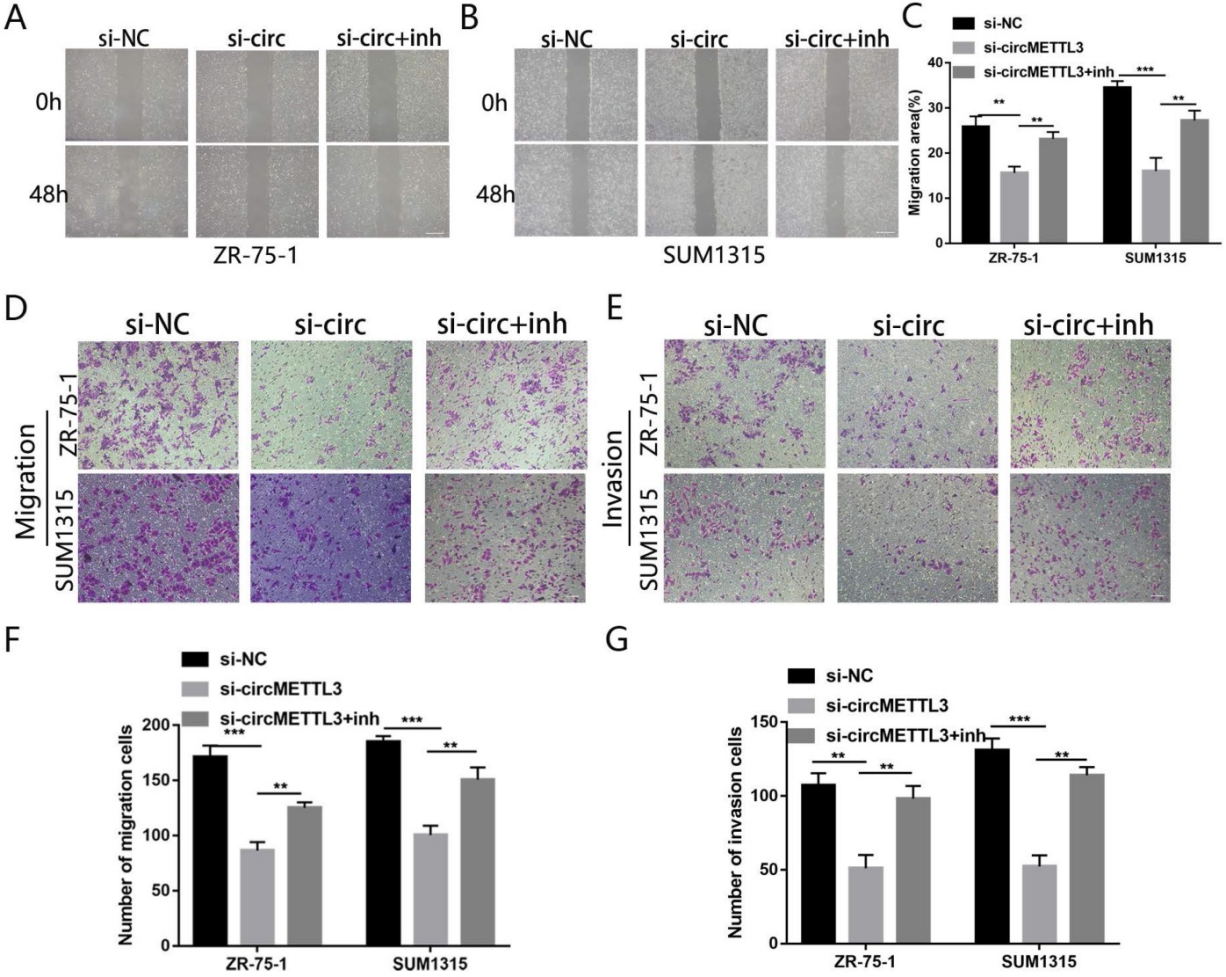
** miR-31-5p partly reverses the metastasis caused by circMETTL3 in breast cancer cells lines.** A-C. The wound healing assays were used to detect the ZR-75-1(A) and SUM1315 (B) cell lines transfected with negative control (si-NC), si-circMETTL3 (si-circ) or circMETTL3+miR-31-5p inhibitor (si-circ+inh) migration ability. Scale bar, 100 µm D-G. Transwell assay was performed to determine the migration (D and F) and invasion (E and G) abilities of ZR-75-1 and SUM1315 cell lines transfected with negative control, si-circMETTL3 or circMETTL3+miR-31-5p inhibitor. Scale bar, 100 µm. Data were shown as mean ± SD, *p<0.01, **p <0.01, ***p<0.001.

We then explored the effect of miR-31-5p on the role of circMETTL3 in breast cancer. The CCK-8 assay showed that inhibition of miR-31-5p expression partly reversed the decrease in breast cancer cells proliferation caused by knockdown of circMETTL3 (Fig. 5A-B). Number of colonies (Fig. 5C) and EdU positive cells also increased significantly (Fig. 5D-E). Moreover, downregulated miR-31-5p could promote migration area inhibited by knockdown of circMETTL3 (Fig. 6A-C). Number of migration and invasion cells increased with inhibition of miR-31-5p (Fig. 6D-G). Overall, these results confirmed circMETTL3 promoted breast cancer progression by acting as a sponge of miR-31-5p.

### CDK1 is a direct target of miR-31-5p in breast cancer

**Figure 7:**
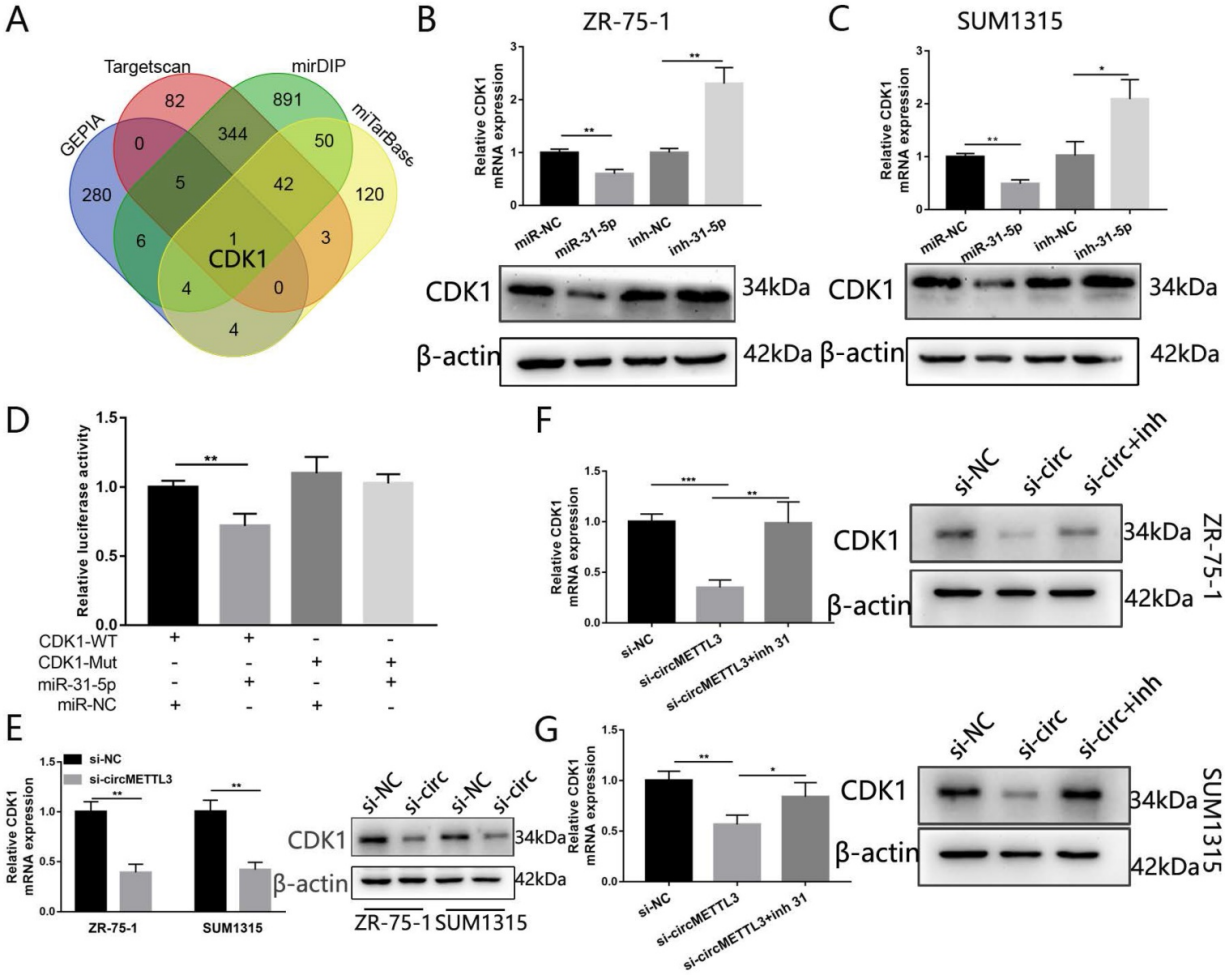
** CDK1 is the target of miR-31-5p and indirectly regulated by circMETTL3.** A. The Venn-diagram indicates the number of genes that overlapped in three publicly available bioinformatics algorithms (Targetscan, mirDIP, miTarbase) and top 300 upregulated genes in breast cancer downloaded from GEPIA (GEPIA). B-C: The mRNA and protein expression levels of CDK1 in ZR-75-1 (B) and SUM1315(C) cell lines transfected with miR-31-5p mimic (miR-31-5p) or miR-31-5p inhibitor (inh-31-5p). D. The relative luciferase activities were detected in 293T cells after transfected with CDK1 3'UTR-WT or CDK1 3'UTR-Mut and miR-31-5p mimics or miR-NC, respectively. E. Expression of CDK1 in breast cancer cells after knockdown of circMETTL3. F-G: qRT-PCR and western blot were excused to detect CDK1 expression in ZR-75-1 (F) and SUM1315 (G) cell lines transfected with negative control (si-NC), si-circMETTL3 (si-circ) or circMETTL3+miR-31-5p inhibitor(si-circ+inh). Data were shown as mean ± SD, *p<0.01, **p <0.01, ***p<0.001.

We used three algorithms (mirDIP, Targetscan and miTarBase) and GEPIA to identity the putative targeted genes of miR-31-5p. The overlap of the targeted genes predicted by the three algorithms and the 300 genes upregulated in breast cancer in GEPIA showed that CDK1 might be the targeted gene of miR-31-5p (Fig. 7A). Forced miR-31-5p expression decreased both CDK1 mRNA and protein expression, while inhibition of miR-31-5p reversed the effect (Fig. 7B-C).We then performed dual-luciferase reporter assays and found upregulated miR-31-5p significantly inhibited luciferase activity of CDK1-WT rather than that of CDK1-Mut (Fig. 7D). Moreover, knockdown of circMETTL3 inhibited CKD1 expression in breast cancer cells (Fig. 7E) while overexpression of circMETTL3 increased CKD1 expression (Fig. S5B-C), but inhibition of miR-31-5p partly reversed the effect (Fig. 7F-G). These results proved that circMETTL3 increased CDK1 expression by sponging miR-31-5p to promote breast cancer progression.

### circMETTL3 is upregulated in a m6A-dependent manner

**Figure 8:**
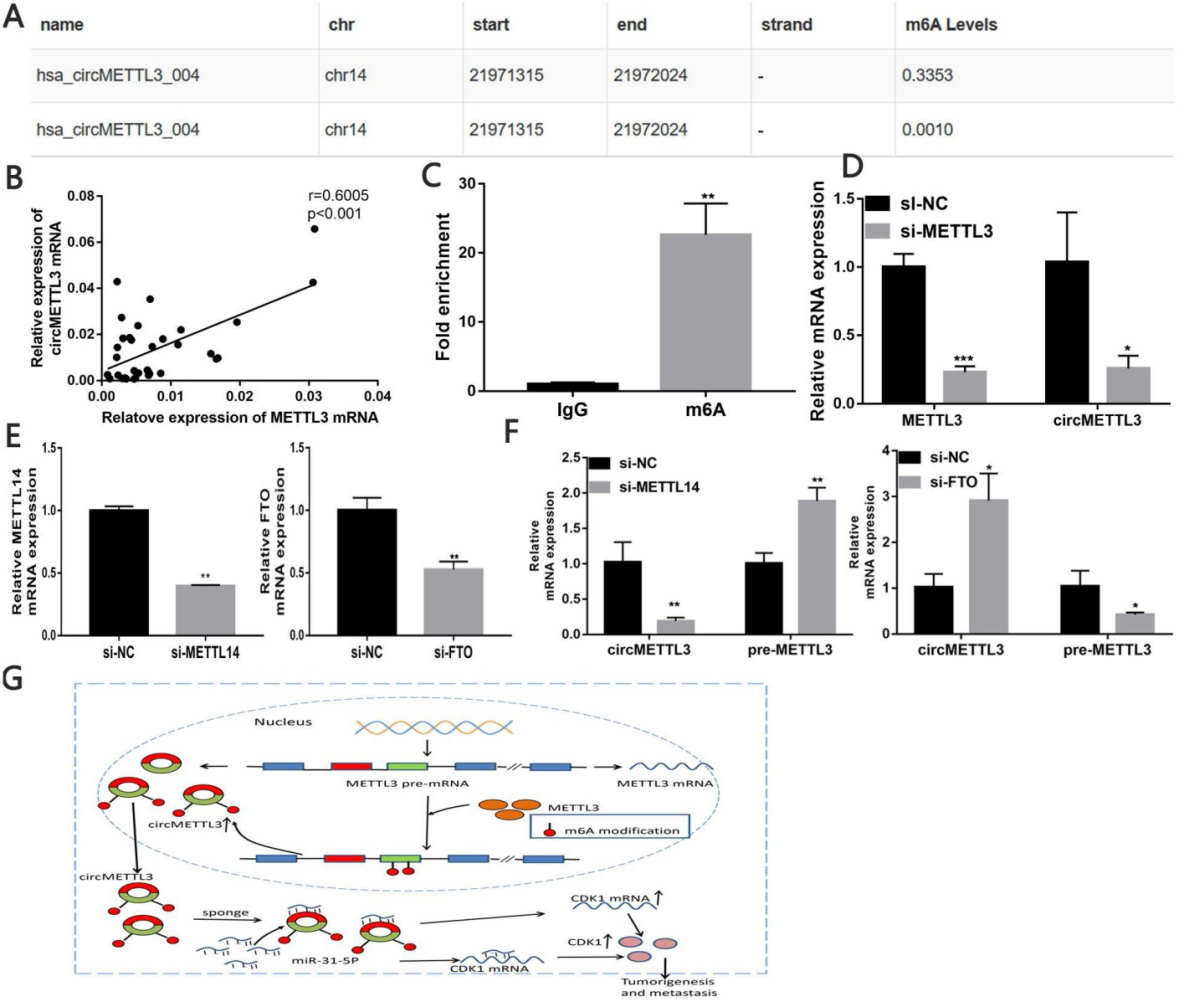
** circMETTL3 is upregulated in a m6A-dependent manner.** A. Possibility of m6A modification of circMETTL3 B. Correlation between circMETTL3 and METTL3 expression in breast cancer tissues. C. Anti-m6A RIP experiments were performed to detect circMETTL3 expression. D. qRT-PCR showed METTL3 and circMETTL3 expression after knockdown of METTL3. E. qRT-PCR analysis of the transfection efficacy of siRNA on Mettl14 and FTO. F. qRT-PCR showed circMETTL3 and pre-METTL3 expression after knockdown of METTL14 or FTO. G. Schematic diagram illustrates the mechanism of circMETTL3 up-regulated in a m6A-dependent manner to promote breast cancer progression through circMETTL3/miR-31-5p/CDK1 axis. Data were shown as mean ± SD, *p <0.05, **p <0.01, ***p<0.001.

Through the circBank, we found that cicMETTL3 might have m6A modification (Fig. 8A). We detected correlation between METTL3 and circMETTL3 expression in breast cancer tissues and found the expression of circMETTL3 and METTL3 is positively correlated (Fig. 8B). MeRIP assay showed circMETTL3 was highly recruited in m6A precipitated fraction (Fig. 8C). Previous studies showed m6As control circ-ZNF609 biogenesis 27. We then explored whether the m6A modification of circMETTL3 affected its expression. The results showed knockdown of METTL3 decreased circMETTL3 expression (Fig. 8D). Moreover, METTL3 increased CDK1 expression in the absence of circMETTL3 (Fig. S6A-B), and the result was consistent with our previous study 21. As circMETTL3 was derived from *METTL3,* we knock downed METTL14 or FTO expression to regulate m6A levels in circMETTL3 to avoid changes in circMETTL3 expression caused by the off-target effects of knockdown of METTL3. Downregulated METTL14 decreased circMETTL3 expression and increased pre-METTL3 expression, while decreased FTO expression caused opposite effect (Fig. 8E-F). Moreover, m6A modification of circMETTL3 increases its expression (Fig. 8G).

## Discussion

In the present study, we characterized a novel circRNA, named as circMETTL3. The circRNA is a 623 nt transcript originated from the exon-2 and exon-3 of METTL3 gene. It was highly expressed in breast cancer cell lines and breast cancer tumor tissues. Moreover, circMETTL3 could promote proliferation and metastasis abilities of breast cancer cells, while knockdown of circMETTL3 showed an opposite effect. Though the cell lines ZR-75-1 and SUM1315 had different estrogen receptor status, circMETTL3 performed oncogenic function in both of the two cell lines, indicating the oncogenic role of circMETTL3 are estrogen receptor status-independent. We then found circMETTL3 mainly located in cytoplasm breast cancer cells and contained miRNA response element of miR-31-5p through bioinformatics analyses. Further, dual-luciferase reporter, anti-AGO2 RIP and RNA pull-down assays confirmed that circMETTL3 could interact with miR-31-5p directly and knockdown of circMETTL3 increased miR-31-5p expression. We also found that miR-31-5p was positively correlated with patients' overall survival based on the TCGA dataset. Through bioinformatics analysis, we identified CDK1 as a potential target of miR-31-5p, which was confirmed by luciferase reporter assay. CDK1, a member of the CDKs, plays a critical role in different types of cancers 21, 28-30. The other study showed CDK1 acted as a novel prognostic indicator for early breast cancer 31. According to our data, downregulated of circMETTL3 decreased CDK1 expression. These findings suggest circMETTL3 acts as an oncogene in breast cancer progression and become a potential diagnostic and therapy target for breast cancer patients. Previous studies showed circRNAs might perform biological functions in tumors through different mechanisms, including act as a sponge of miRNAs 32, interact with RNA binding proteins, enhance targeted gene mRNA stability, influence host gene, or encode peptides 10, 33. For example, circANKS1B promoted breast cancer metastasis via acting as a sponge of miRNAs 12. circBMPR2 inhibited breast cancer progression via circBMPR2/miR-553/USP4 axis 34. circNSUN2 interacted with YTHDC1 to promote its cytoplasmic export and stabilized HMGA2 mRNA via interacting with IGF2BP2 35. circKIAA1429 accelerated hepatocellular carcinoma advancement through enhancing ZEB1 mRNA stability 26. circSMARCA5 terminated the transcription of SMARCA5 to inhibit DNA damage repair 14. circSHPRH suppressed glioma tumorigenesis by encoding a novel protein 36. circFNDC3B encoded a novel protein to inhibit colon cancer progression 37. In our study, results illustrated circMETTL3 acts as a sponge of miR-31-5p to increase CKD1 expression, in line with previous studies 11, 12, 38.

Previous studies showed circRNAs might act as a sponge to increase its host gene expression 39-41 or decrease the parental gene expression by terminating the transcription of the parental gene 14. However, the relation of circMETTL3 and its parental gene, METTL3, was still elusive. Several studies showed METTL3 promote different cancers through affecting its target genes and non-coding RNAs in an m6A dependent manner 24, 25, 42, 43. We then explored whether METTL3 affected circMETTL3 expression by the mechanism of m6A modification. As circMETTL3 was derived from* METTL3*, we knockdown METTL14, which helps METTL3 to recognize its RNA substrates, or FTO, function as m6A demethylases, to regulate m6A levels in circMETTL3 to avoid changes in circMETTL3 expression caused by the off-target effects of knockdown of METTL3. We found that downregulated METTL14 could decrease circMETTL3 expression and increase pre-METTL3 expression, while decreased FTO expression caused opposite effect. These results indicated m6A modification might affect circMETTL3 expression, in line with previous study METTL3 affect circ-ZNF609 biogenesis in an m6A dependent manner 27. We also found that knockdown of circMETTL3 expression with small interfering RNA could not affect the parental gene METTL3 expression. Moreover, the expression of METTL3 and circMETTL3 are positively correlated in breast cancer tissues. All the results suggested that METTL3 might increase circMETTL3 expression in an m6A dependent manner. It illustrated METTL3 increased circMeETTL3 expression, derived from its own, to promote breast cancer progression by its methyltransferase activity 24, 25. Another component in m6A methyltransferase enzymes, KIAA1429 and its circKIAA1429 were also highly expressed and promoted hepatocellular carcinoma advancement 26, 44, showing the similar mode as METTL3 and circMETTL3 in breast cancer. Moreover, parental gene could regulate corresponding circRNAs expression through their own function, besides acting as sponges of miRNAs or terminating the transcription of the parental gene, providing a new insight into the relationship between circRNAs and their parental gene.

## Conclusion

In conclusion, our results illustrated the vital role of circMETTL3 in the breast cancer progression. Mechanistically, circMETTL3 harbors miR-31-5p to upregulate CDK1 expression. Moreover, modification of circMETTL3 increases its expression. METTL3, the circMETTL3 host gene, is the most important part of methyltransferase complex and may increase circMETTL3 expression in an m6A-depenent manner. Thus, circMETTL3 might act as a potential therapeutic target for breast cancer.

## Supplementary Material

Supplementary figures and tables.

## Abbreviations

CircRNA: Circular RNA
m6A: N6-methyladenosine
qRT-PCR: Real-time quantitative polymerase chain reaction
cDNA: Complementary DNA
gDNA: Genomic DNA
3'UTR: 3′ -untranslated region
ceRNA: Competing endogenous RNA
FISH: Fluorescence *in situ* hybridization
miRNA: MicroRNA
RIP: RNA immunoprecipitation
RNAi: RNA interfere
