# Supplementary material for: CircMETTL3, upregulated in a m6A-dependent manner, promotes breast cancer progression
Source: Int J Biol Sci. 2021 Mar 15;17(5):1178–90. doi: 10.7150/ijbs.57783 (PMC8040468; doi:10.7150/ijbs.57783)
Supplement: Supplementary file 1 — Supplementary figures and tables. [file ijbsv17p1178s1.pdf]

Table S1: The primer sequence of qRT-PCR

circMETTL3	F: 5’-GCCAAGAAATCAAGGAAA-3’
	R: 5’-GCACTGGGCTGTCACTA-3’
METTL3	F: 5’-CACAGAGTGTCGGAGGTGATTC-3’
	R: 5’-CCTGTAGTACGGGTATGTTGAGC-3’
CDK1	F: 5’-CTCTGATTGGCTGCTTTGAA-3’
	R: 5’-TTTCATGGCTACCACTTGACC-3’
METTL14	F:5’-TTGATGAGATTGCAGCACCT-3’
	R:5’-TGCTACGCTTCACAGTTCCTT-3’
FTO	F:5’-CGAGAGCGCGAAGCTAAGA-3’
	R:5’-GCTGCCACTGCTGATAGAAT-3’
pre-METTL3	F:5’-TCACGGTCAGTGTCTTATTG-3’
	R:5’-AGAACGAGGGGAGGTATGGG-3’
β -actin	F:5’-AGCGAGCATCCCCCAAAGTT-3’
	R:5’-GGGCACGAAGGCTCATCATT-3’

Table S2: siRNA and RNA oligonucleotides sequences.

名称	Sense（5’-3’）
si-circMETTL3-1	GUCUCGUUCUUCUAGAUGCTT
si-circMETTL3-2	UUGUCUCGUUCUUCUAGAUTT
miR-31-5p mimics	ACGCAAGAUGCUGGCAUAGCU
mimics control	UUCUCCGAACGUGUCACGUTT
miR-31-5p inhibitor	AGCUAUGCCAGCAUCUUGCCU
Inhibitor control	CAGUACUUUUGUGUAGUACAA
si-METTL14	GGCUAAAGGAUGAGUUAAUTT
si-FTO	GCAGCAUACAACGUAACUUTT

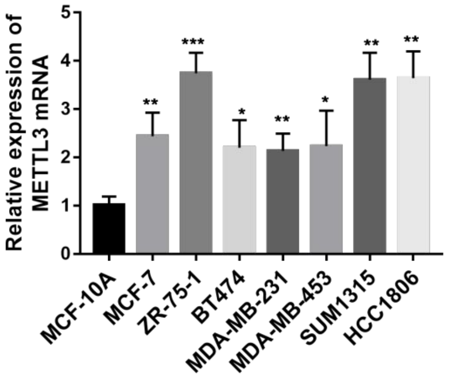
Relative expression of METTL3 mRNA
5
4
3
2
1
0
**
***
*
**
*
**
**
MCF-10A
MCF-7
ZR-75-1
BT474
MDA-MB-231
MDA-MB-453
SUM1315
HCC1806

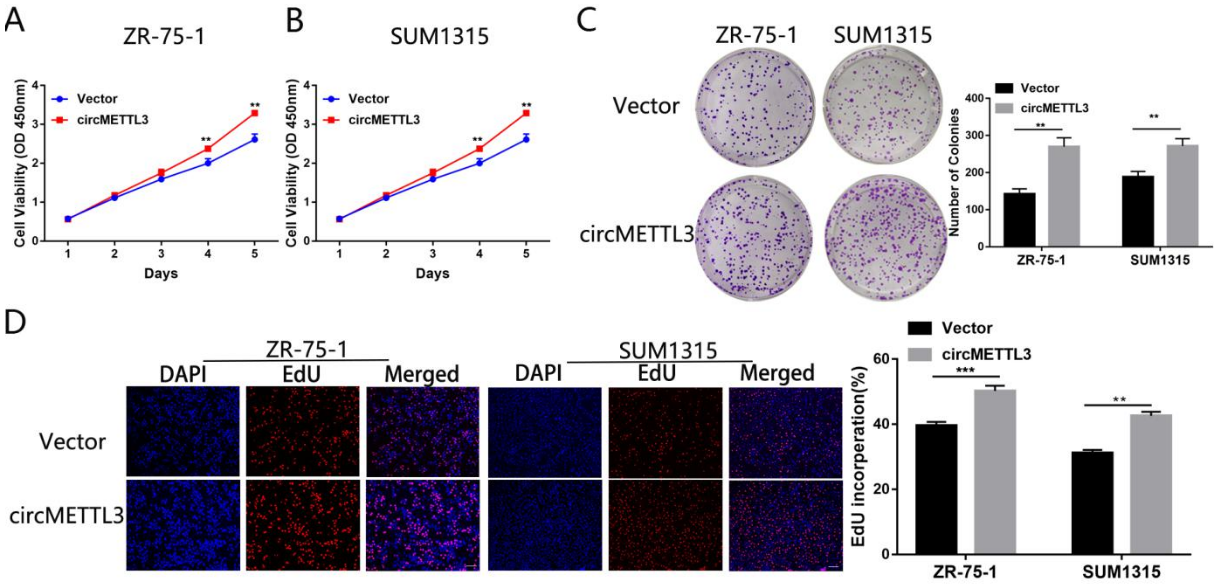
A
ZR-75-1
Cell Viability (OD 450nm)
Vector
circMETTL3
Days
B
SUM1315
Cell Viability (OD 450nm)
Vector
circMETTL3
Days
C
ZR-75-1
SUM1315
Vector
circMETTL3
Number of Colonies
Vector
circMETTL3
ZR-75-1
SUM1315
D
ZR-75-1
DAPI
EdU
Merged
SUM1315
DAPI
EdU
Merged
Vector
circMETTL3
Vector
circMETTL3
EdU incorperation(%)
ZR-75-1
SUM1315

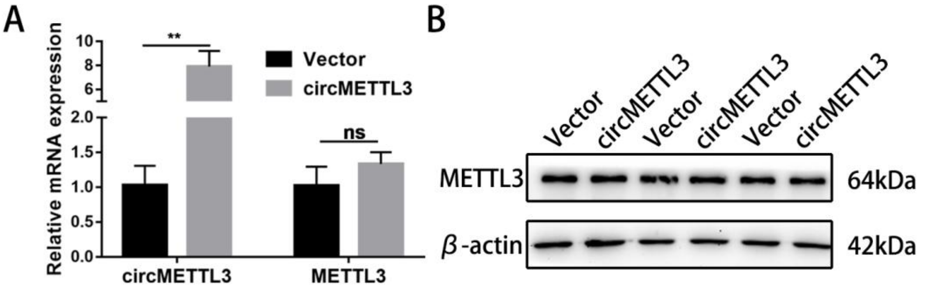
A
Relative mRNA expression
Vector
circMETTL3
**
ns
circMETTL3
METTL3
B
Vector
circMETTL3
Vector
circMETTL3
Vector
circMETTL3
METTL3
64kDa
β-actin
42kDa

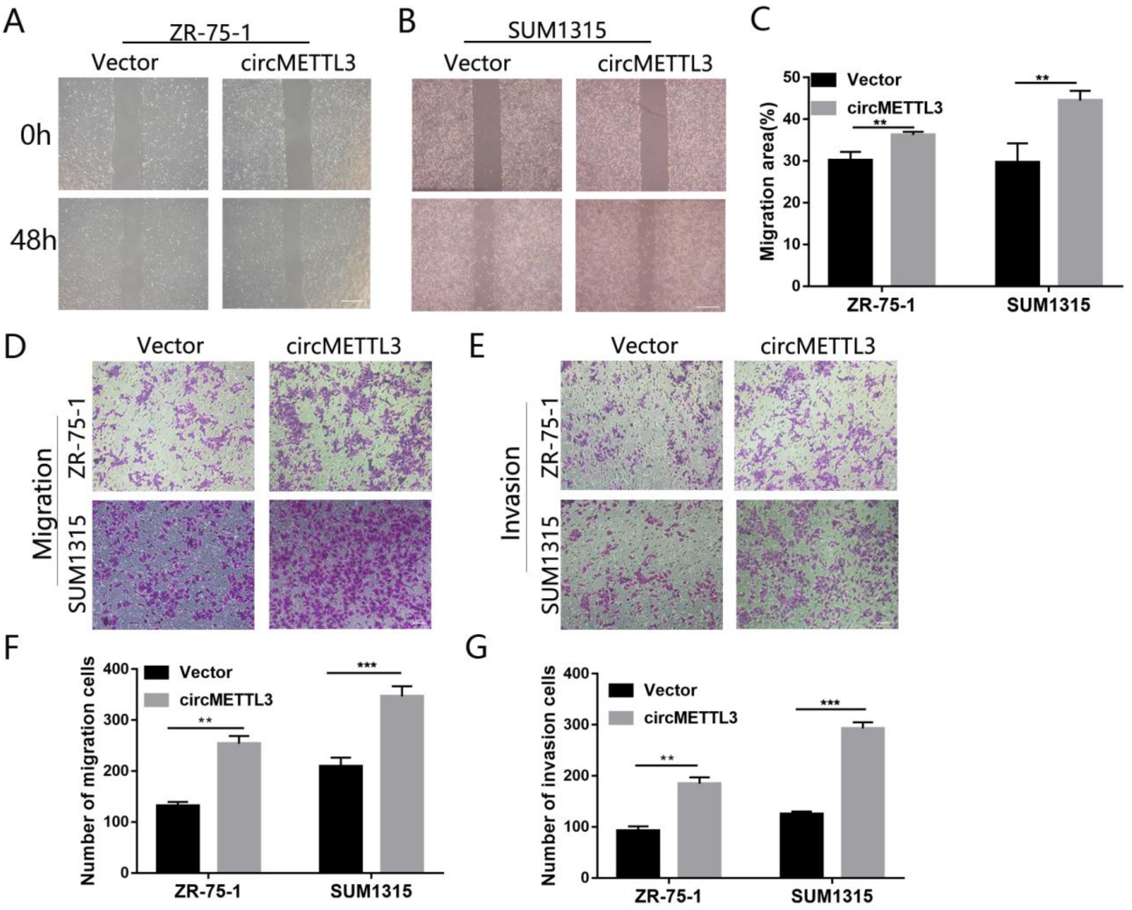
A
ZR-75-1
Vector
circMETTL3
0h
48h
B
SUM1315
Vector
circMETTL3
C
Vector
circMETTL3
Migration area(%)
50
40
30
20
10
0
**
**
ZR-75-1
SUM1315
D
Vector
circMETTL3
Migration
ZR-75-1
SUM1315
E
Vector
circMETTL3
Invasion
ZR-75-1
SUM1315
F
Vector
circMETTL3
Number of migration cells
400
300
200
100
0
**
***
ZR-75-1
SUM1315
G
Vector
circMETTL3
Number of invasion cells
400
300
200
100
0
**
***
ZR-75-1
SUM1315

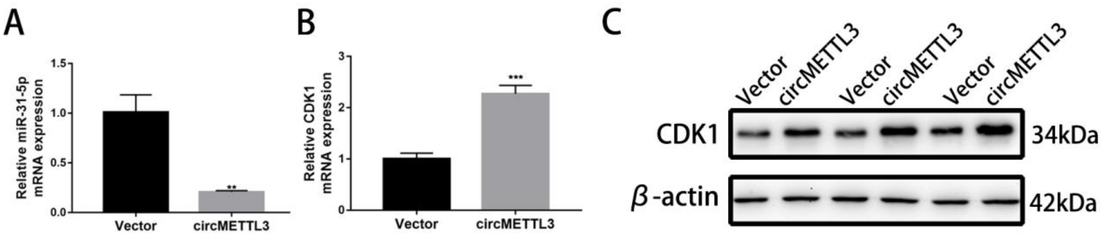
A
Relative miR-31-5p mRNA expression
1.5
1.0
0.5
0.0
**
Vector
circMETTL3
B
Relative CDK1 mRNA expression
3
2
1
0
***
Vector
circMETTL3
C
Vector
circMETTL3
Vector
circMETTL3
Vector
circMETTL3
CDK1
34kDa
β-actin
42kDa

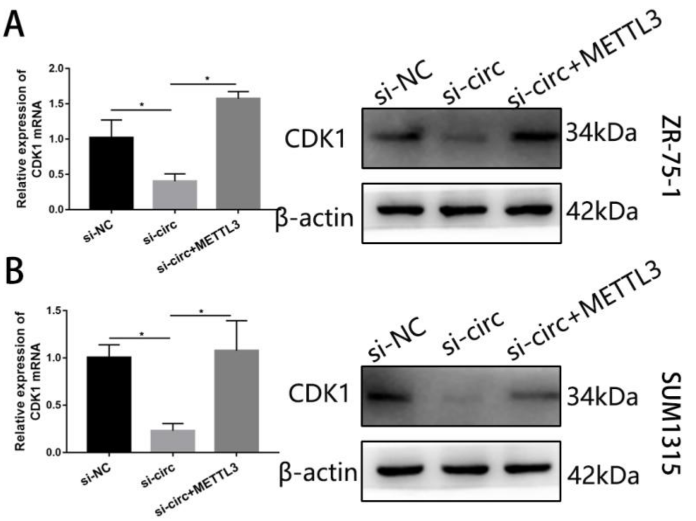
A
Relative expression of CDK1 mRNA
2.0
1.5
1.0
0.5
0.0
*
*
si-NC
si-circ
si-circ+METTL3
si-NC
si-circ
si-circ+METTL3
CDK1
34kDa
β-actin
42kDa
ZR-75-1
B
Relative expression of CDK1 mRNA
1.5
1.0
0.5
0.0
*
*
si-NC
si-circ
si-circ+METTL3
si-NC
si-circ
si-circ+METTL3
CDK1
34kDa
β-actin
42kDa
SUM1315

Figure S1: Expression of circMETTL3 in the breast cancer cell lines and MCF-10A by qRT-PCR. Data were shown as mean ± SD, *p <0.05, **p <0.01, ***p<0.001.

Figure S2: A-B: The growth curves of ZR-75-1 (A) and SUM1315 (B) cell lines transfected with indicated vectors were evaluated by CCK-8 assays. C-D: Cell proliferation was determined by colony formation (C) and EdU assays (D) in ZR-75-1and SUM1315 cell lines transfected with indicated vectors. Data were shown as mean ± SD, *p <0.05, **p <0.01, ***p<0.001.

Figure S3: A-B: Overexpression of circMETTL3 had no effect on METTL3 mRNA expression (A) and protein level (B) in the tumors of mice treated with vector or overexpression-circMETTL3. Data were shown as mean ± SD, *p <0.05, **p <0.01.

Figure S4: A-C: The wound healing assays were used to detect the cell migration ability after transfecting breast cancer cells. Scale bar, 200 μm. D-G: Migration (D and F) and invasion (E and G) abilities of breast cancer cells transfected with indicated vectors were determined by transwell assay. Scale bar, 100 μm. Data were shown as mean ± SD, *p <0.05, **p <0.01, ***p<0.001.

Figure S5: A-B: Overexpression of circMETTL3 decreased expression of

miR-31-5p (A) and increased CDK1 expression (B-C) in in the animal tumors. Data were shown as mean ± SD, *p <0.05, **p <0.01, ***p<0.001.

Figure S6: A-B: METTL3 increased CDK1 expression in the absence of circMETTL3 in ZR-75-1 (A) and SUM1315 (B). Data were shown as mean ± SD, *p <0.05.
